# Report on Practical Medicine

**Published:** 1875-03

**Authors:** Norman Bridge

**Affiliations:** Chicago


### Progress in Medical Sciences.

ARTICLE I.

### REPORT ON PRACTICAL MEDICINE.

BY NORMAN BRIDGE, M.D., CHICAGO.

1. Hodgkin's Disease. By W. H. TRIPLETT, M.D. (*Richmond and Louisville Med. Jour.*, April 1, 1874.)

2. Cerebral Rheumatism. By Prof. J. M. DA COSTA. (*Am. Jour. Med. Sciences*, Jan., 1875.)

3. Parotiditis in Acute Diseases. By Prof. CROCQ. (*Jour. de Med.; Med. Times & Gaz.*)

4. The Hypodermic Injection of Atropia in Sun Stroke. By J. R. BARNETT, M.D. (*Am. Jour. Med. Sciences*, Jan., 1875.)

5. Muriatic Acid in the Treatment of Typhoid Fever. By S. K. CRAWFORD, M.D. (*Transac. Ill. State Med. Soc.*, 1874.)

1. Dr. Triplett read a paper giving an account of two additional cases of Hodgkin's disease, and reviewing the literature of the subject. The first case was in a woman forty years old. She had presented herself five years before, with large painful tumors of the right mamma; they were enlarged lymphatic glands, but so resembled carcinoma that it was diagnosed as such. In fifteen months these tumefactions had disappeared, and appeared "under her arms and all over." "Enormous tumors occupied the axillæ, growing over and around the anterior walls; her neck was full of small ones." There were small tumors along the arm, and large ones in the inguinal regions. None of the tumors suppurated; "they were soft, elastic, painless." The patient was still living, said the doctor, "a year ago." The other case was in a woman sixty-five years old, who had always enjoyed good health until 1868, when she had pneumonia, making a speedy recovery; she had afterward eczema and sore nose in winter. "Fifteen months ago she noticed small lymphatic tumors in the neck." Soon after one appeared in the right axilla, which subsequently disappeared. In the winter of 1872–73, she had another pneumonia, making a slow recovery, and in the following spring, "there was an explosion; the diseased glands suddenly showing themselves all over the body;" "they rapidly grew to immense size." At the writing of the paper the abdomen was tumid and tender to touch, but the liver was not enlarged, neither was the spleen; there was no cramp, diarrhœa, constipation, or dyspepsia; the appetite and digestion were good. Indeed, all the physiological functions were normally performed, with the exception that there was slight dyspnœa, although by auscultation no abnormal sound further than slight mucous rales could be demonstrated.

The enlarged glands were of all sizes, from that of an almond to that of a turkey's egg. The red corpuscles of the blood were not decreased—the white ones were slightly increased; the blood was not leucocythæmic. He insists on the characteristics of Hodgkin's disease about as follows: It is not connected with any known diathesis; it begins by slight enlargement of a few lymphatic glands, chiefly those of the neck, this stage lasting a few months or years. This is followed by an explosion, when it seems that almost or quite every gland in the body becomes suddenly enlarged. The physiological functions remain long unaffected, and the patient dies by the exhaustion largely due to mechanical pressure. No treatment is of any avail.

2. Prof. D. applies the term cerebral rheumatism to all cases of acute rheumatism that are accompanied by such decided cerebral symptoms as appear to constitute the real feature of the disease. He records, as illustrative of his subject, twelve cases.

In the first, after apparent convalescence, Bright's disease appeared, followed by hebetude, stupor, suppression of urine, and death.

In case 2, the cerebral symptoms occurred during a relapse of joint affection, stupor and delirium occurred, albuminous urine with granular and oily casts, and death.

In case 3, stupor occurred in the course of a mild acute rheumatism ; there was no heart trouble ; death resulted.

In case 4, there was marked delirium of hysterical type, with no headache or high temperature. Death occurred from exhaustion in the ninth week of the disease.

Case 5 was one of acute rheumatism, with pericarditis, restlessness and delirium, with recovery.

Case 6 had endo-pericarditis, delirium and stupor, but without headache, and resulted in death.

Case 7 had pericarditis, delirium, muscular tremors, gradual sinking, and ended in death.

In case 8 there were restlessness and hysterical manifestations preceding stupor ; headache, spasmodic contraction of facial muscles, coma, and before death, a temperature of 108 1-5 degrees.

Case 9 had delirium, one sided facial palsy ; slight motor paralysis of muscles of arm and leg of same side, and gradual and complete recovery.

Case 10 was one of cerebral rheumatism, terminating in a typhoid condition, with no cardiac and slight joint trouble. The temperature was not over 102 degrees, and death resulted in sixty-five days from the beginning of the attack.

Case 11 was one of acute rheumatism, with no cerebral or heart symptoms, but with typhoid condition and recovery ; yet at one time the temperature reached 110 degrees Fahrenheit.

Case 12 had marked delirium and stupor, coma, apparent death, and recovery under inhalations of ammonia and stimulants.

The only autopsies procurable in these cases revealed : in case 2, no cerebral lesion ; in case 3, soft coagula in arteries of the base of the brain, but no firm clots ; in case 4, the vessels of the choroid plexus highly congested, the branches of the middle meningeal arteries, many of them, occluded by fibrinous plugs.

In commenting on the pathology of the affection he was struck with the slight thermometric fluctuation. His observations negative the commonly accepted doctrine, that "hyperpyrexia in acute rheumatism and brain symptoms are synonymous."

He believes that leaving out exceptional cases of real rheumatic meningitis, the true pathology of the cerebral disorder is to be found in the action of rheumatic poison on the brain ; but that this poison may fasten on the lining membrane of the small cerebral vessels and lead to plugging of them. Occasionally an embolus from the heart is washed to the brain and plugs up a vessel, but more usually such plugging is due to thrombi there formed. Hyperpyrexia results where the rheumatic poison acts on the *nerve centres regulating temperature.*

The idea that quinia or other drug, in medicinal doses, has any power to produce this disorder, he believes nonsensical. Cerebral rheumatism is much more common some years than others, but our author can find no meterological condition to account for the fact.

Cases occurring suddenly, formerly called apoplectic, are, he thinks, mostly uræmic, and he insists on the urine always being examined.

3. Prof. C. has carefully studied the subject of parotiditis as a sequence of acute disease, and his conclusions as to its nature and causation differ from those of many authors. In his opinion it is not due to pyæmia, but occurs in those diseases wherein there is stomatitis, and as a result of this. The inflammation of the mucous membrane of the mouth travels up the duct to the gland. When the soreness of the gland is first noted, and long before any suppuration can have taken place in the mass of the latter, purulent matter may be squeezed from the mouth of the duct. In the advanced stage of typhoid or typhus fevers, in rubeola, variola and scarlatina, as well as in the reaction of cholera, stomatitis is either a distinctive or a frequent occurrence; it is in these diseases the disorder in question most often occurs. He claims that the parotiditis never precedes the stomatitis. The idea of the old authors, of a "critical" and a "symptomatic" parotiditis, is a mistake.

4. Dr. B. urges the use of atropia in insolation, on the ground that it is "the most general excitant of the vital nervous centres available;" he claims advantage for it hypodermically, because it is available irrespective of gastric conditions—by this means the danger of non-absorption is avoided; and he claims that "like other stimuli, it is indicated in by far the greater proportion of sunstroke cases," while "it is permissible in many cases not amenable to other excitants." It is a cardiac stimulant, and at the same time by its vaso-motor stimulation leads to contraction of any over-distended vessels of the brain, therein avoiding a danger many other stimuli are liable to, of increasing any cerebral congestion.

The doctor reports two cases wherein this treatment was used that fairly seem to vindicate his theory. His doses were from 1-60 to 1-40 gr. The treatment deserves trial.

5. Dr. Crawford reports four series, of ten cases each, of typhoid fever. The first series was treated with alteratives, turpentine, egg-nog, diuretics, with leeches, spongings, etc., locally. There were six deaths. In the second series the treatment was stimulants, nutritious diet, and Dover powder. Three deaths resulted. In the third group the treatment consisted in alteratives, anodynes, stimulants, nutritious diet, strychnia, nitric acid, turpentine emulsion and diuretics. Two cases were fatal. None of the treatment in any of the preceding cases having had the slightest effect in lessening the "dead epithelium" on the "surface of the digestive tube," the fourth group of cases was treated with dilute muriatic acid, beef tea and milk, with recovery in every case. In all these ten cases there was an absence of diarrhœa, suppression of urine, pneumonic complications, and indeed all other complications. The tongue was cleaned of its coating in an average of fifty-one hours from the commencement of the acid treatment, and with one or two exceptions remained clean during the course of the disease.

He believes quinine aggravates the disease in all cases uncomplicated with malaria. He avers that in two cases where, during the second week of the disease, the tongue was clean and moist, he tried the experiment of giving ½ gr. doses of *quinia sulphas.* The result was that in both cases "a dry and irritable condition of the tongue" was induced within twenty-four hours. He believes muriatic acid is "*the best* remedy known" for typhoid fever.

